# Anti-Inflammatory and Immunoregulatory Functions of Artemisinin and Its Derivatives

**DOI:** 10.1155/2015/435713

**Published:** 2015-04-16

**Authors:** Chenchen Shi, Haipeng Li, Yifu Yang, Lifei Hou

**Affiliations:** ^1^Laboratory of Immunology and Virology, Shanghai University of Traditional Chinese Medicine, Shanghai, China; ^2^Department of Orthopedics, Beijing Army General Hospital, Beijing, China; ^3^Program in Cellular and Molecular Medicine, Boston Children's Hospital and Department of Pediatrics, Harvard Medical School, Boston, MA 02115, USA

## Abstract

Artemisinin and its derivatives are widely used in the world as the first-line antimalarial drug. Recently, growing evidences reveal that artemisinin and its derivatives also possess potent anti-inflammatory and immunoregulatory properties. Meanwhile, researchers around the world are still exploring the unknown bioactivities of artemisinin derivatives. In this review, we provide a comprehensive discussion on recent advances of artemisinin derivatives affecting inflammation and autoimmunity, the underlying molecular mechanisms, and also drug development of artemisinins beyond antimalarial functions.

## 1. Introduction

**Figure 1:**
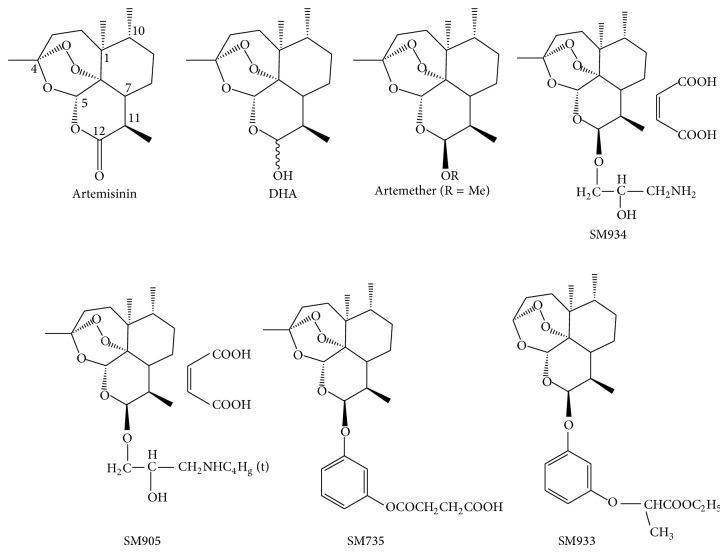
Chemical structure of artemisinin and its derivatives.

Artemisinin was isolated from* Artemisia annua *L. in 1972 by Chinese researchers. At the end of 1975, its unique chemical structure was elucidated, as a sesquiterpene lactone bearing a peroxy group, quite different from that of all known antimalarial drugs (reviewed in [1]). Artemisinin and its derivatives are currently considered the most effective drug in treating cerebral malaria and chloroquine resistant falciparum malaria [2, 3]. It is also recognized as the “best hope for the treatment of malaria” by the World Health Organization because of its effectiveness, nonresistant characteristics, and minimal side effects [2, 3]. The active metabolite of artemisinin is dihydroartemisinin (DHA). Currently, artemisinin derivatives used in clinical treatment include DHA, artemether, artesunate, and arteether. In addition to their excellent antimalarial effects, the clinical and experimental studies also suggested that artemisinin and its derivatives possess potent immune-suppressive abilities to treat autoimmune and allergic diseases. Recently, scientists from Shanghai Institute of Materia Medica (SIMM, CAS) designed a series of novel artemisinin derivatives with lower toxicity, higher bioavailability, and potent immunosuppressive activity. In this paper, we will review the progress of the anti-inflammation and immunoregulatory studies of artemisinin family compounds including both commercial available and newly synthesized ones including 3-(12-*β*-artemisininoxy) phenoxyl succinic acid (SM735) [4], 1-(12-*β*-dihydroartemisinoxy)-2-hydroxy-3-tert-butylaminopropane maleate (SM905) [5–7], ethyl 2-[4-(12-*β*-artemisininoxy)] phenoxylpropionate (SM933) [8], and 2′-aminoarteether (*β*) maleate (SM934) [9–12] (chemical structures were shown in Figure 1). Of note, the SM934 is recently approved by the China Food and Drug Administration for clinical trial as novel therapeutic agent to treat systemic lupus erythematosus (SLE).

## 2. Antimalarial Mechanism of Artemisinin

Artemisinin is a sesquiterpene lactone containing peroxide bridge. Studies have shown that peroxide bridge plays an essential role in antimalarial pathways for artemisinin. The damage or absence of such peroxide bridge will reduce or even diminish the antimalarial effect. In contrast, leaving peroxide bridge untouched, antimalarial effect of artemisinin remains, no matter how the sesquiterpene was chemically modified [13, 14].

To date, it is broadly accepted that artemisinin exerts antimalarial effects through the following molecular mechanisms: Heme or free iron will break the peroxide bridge, which results in the degradation of the molecular structure of artemisinin to form the nucleophilic radical metabolites with the center of C_4_. Consequently, the free radicals, acting as an alkylating agent, will attack macromolecular bearing electrophilic groups or centers, which eventually leads to parasitic death [14, 15]. Actually, the red blood cells have high level of oxidative stress once they are infected with* Plasmodium*. Meanwhile, the intracellular free radicals and lipid peroxidation level will dramatically increase due to oxidative stress. As a result, parasite-infected red blood cells will increase their susceptibility to artemisinin. Accordingly,* in vivo*, artemisinin is effective in killing parasite-infected red blood cells at nM levels; in sharp contrast, artemisinin only shows marginal effects on resting red blood cells, even with the concentration as high as mM levels [16, 17].

## 3. Anti-Inflammation and Immunoregulatory Effect of Artemisinin

### 3.1. *In Vitro* Immunosuppressive Activity

T cells play pivotal role in acquired immune reaction, which includes three fundamental steps [18, 19]. First, TCR cross-linking drives T cells from G0 to G1 transition and subsequent secretion of T cell growth factor IL-2 and expression of high-affinity receptor IL-2R*α* chain (CD25). Second, through autocrine/paracrine proliferative loop, IL-2 induces clone expansion and maintains survival of activated T cells. Third, after successful clearance of the pathogen, the stimulus for cytokines production is lost and activated T cells thus will undergo apoptosis. However, in autoimmune diseases, due to the persistence of autoantigen, autoreactive T cells will be activated and survive better. Autoreactive T cell proliferation is involved in the pathogenesis of various autoimmune diseases, such as rheumatoid arthritis (RA) and multiple sclerosis (MS) [20, 21]. Artemether is a potent antimalarial drug [1]. In 2007, Wang et al. found artemether significantly suppressed the proliferation and IL-2 and interferon-*γ* (IFN-*γ*) production of T cells triggered by TCR engagement [22]. Artemether significantly inhibited TCR engagement-triggered MAPKs signaling pathway including phosphorylation of ERK1/2, Jnk, and P38. Authors further dissected that artemether majorly affected the function of T cells, rather than the antigen-presenting cells (APCs) to exert the immunosuppressive effects.

In recent years, by inserting new groups to the parent structure of artemisinin, Li from SIMM synthesized a series of artemisinin derivatives with higher water solubility and lower toxicity [23–25]. The new compounds were screened for* in vitro* immunosuppressive activity, majorly focused on suppressing T cell activation. SM735, one of artemisinin derivatives developed by Li group, substantially inhibited the proliferation and IFN-*γ* production of mitogen Con A-stimulated splenocytes [4]. It also significantly suppressed the IL-12, IFN-*γ*, and IL-6 productions from LPS-stimulated splenocytes. SM934 and SM905 were recently synthesized derivatives by Li group in SIMM [23, 25]. Similar to SM735, the studies in Zuo group in SIMM found SM905 possessed potent immunoregulatory properties [5–7].

However, SM934 is quite distinct [11]. Similar to SM905 and artemether,* in vitro*, SM934 significantly inhibited the proliferation and IFN-*γ* production of splenocytes or purified CD4+ T cells induced by mixed lymphocyte reaction (MLR) or TCR cross-linking. In sharp contrast to all of SM905, SM735, and artemether, SM934 exerted no influence on IL-2 production and CD25 upregulation of T cells but could remarkably suppress IL-2-mediated proliferation and survival of activated T cells, which might be the consequence of blocking IL-2-induced phosphorylation of Akt. In addition, through combined staining of CD69 and annexin V, SM934 was found to preferentially promote activated T cells into early apoptosis, leaving resting T cells untouched.

Moreover, there are also studies suggesting that artemisinin derivatives will bind to calmodulin to inhibit phosphodiesterase activity, which causes the increase of intracellular cAMP level, and thus to exert the immunosuppressive activity [26, 27].

### 3.2. Artemisinin Derivatives Treat Rheumatoid Arthritis

Collagen-induced arthritis (CIA) is a classic murine model of human rheumatoid arthritis. In CIA model, SM905 could dramatically prevent or treat arthritis, which was manifested by significantly reduced incidence, joint synovial injury, and inflammatory factors secretion [7]. In addition, oral treatment of SM905 skewed the T cell subset from pathogenic Th17 to protective Th2 subset in CIA model. SM905 treatment increased IL-4 production from T cells and suppressed the ROR*γ*t mRNA expression and IL-17 production from T cells.

K/BxN mice spontaneously develop an autoimmune arthritis disease with many clinical, histopathological, and immunological features of the human rheumatoid arthritis. Breakdown of T and B cells tolerance leads to the production of high-titer autoantibodies against glucose-6-phosphate isomerase (GPI), which can directly induce joint pathology. Given the well-studied disease mechanisms and clearly defined roles of various immune cells, K/BxN mice have been an informative model to investigate therapeutic agents targeting antibody-mediated autoimmune diseases. A recent study by Hou et al. demonstrated that artemisinin analog artesunate remarkably ameliorated the arthritis in K/BxN mouse [28]. Artesunate treatment prevented the arthritis development in young K/BxN mice by inhibiting germinal center formation and production of autoantibodies. In adult K/BxN mice with established arthritis, artesunate treatment rapidly diminished germinal center B cells in a few days. However, artesunate did not affect the follicular helper T cells (Tfh). In contrast to the spontaneous K/BxN model, artesunate treatment exerted minor influence on K/BxN serum transfer induced arthritis, suggesting that artesunate has minor effects on inflammatory responses downstream of antibody production. Thus, authors demonstrated that highly proliferative GC B cells were the most sensitive cellular targets to artesunate treatment. Besides, in rat model of Freund's complete adjuvant-induced arthritis, artesunate was found to produce dose-dependent reduction in joint inflammation and improvement in functional parameters, including stair climbing ability, motility, and suppression of mechanical allodynia [29, 30].

In addition to the animal model, artemisinin derivatives also showed promising effects on human rheumatoid arthritis.* In vitro*, artesunate could significantly inhibit IL-1*β*, IL-6, and IL-8 production from synovial cells of RA patients, when stimulated with TNF-*α*. Further studies demonstrated that artesunate inhibited Akt phosphorylation and I*κ*B degradation by blocking PI3K/Akt signaling pathway downstream of TNF-*α* [30, 31].

### 3.3. Artemisinin Derivatives Treat Systemic Lupus Erythematosus

Systemic lupus erythematosus (SLE) is a chronic autoimmune disease characterized by abnormal accumulation of autoreactive T lymphocytes and production of autoantibody against self-antigen, which result in the development of immune complex-mediated glomerulonephritis and renal failure. Female MRL/lpr mice and NZBW/F1 mice were two well-established murine models for lupus study and represent human lupus disease closely [32]. Hou et al. showed SM934 is able to significantly prolong the lifespan and limit the glomerulonephritis in the MRL/lpr mice by inhibiting both Th1 and Th17 responses [10]. The therapeutic properties of SM934 were characterized by suppressing the serum level of pathogenic cytokines interferon-*γ* (IFN-*γ*) and interleukin-10 (IL-10), reducing the secretion and deposition of pathogenic anti-dsDNA IgG autoantibodies in serum and kidneys, and ameliorating the renal injury. SM934 treatment also rectified the abnormal lymphocyte development including reduced double negative T cells (DN T) and elevated conventional single positive T cells and B cells in the spleen. In addition, SM934 treatment reduced the proportion of CD4^+^CD44^+^CD62L^−^ Teff in spleens. Further investigations revealed that SM934 treatment significantly suppressed the excessive activation of STAT1, STAT3, and STAT5 in lupus.

In NZB/W F1 mice, the* fas* gene is intact, which makes the pathogenesis of NZB/W F1 mice largely different from that of MRL/lpr mice [33]. However, SM934 also exerts comprehensive therapeutic effects on NZB/W F1 mice both in short-term and long-term treatment [9]. Similarly to MRL/lpr mice, SM934 treatment could significantly increase Treg percentage and suppress the Th1 and Th17 responses in NZB/W F1 mice. Clinical improvement was accompanied with decreased Th1-related anti-dsDNA IgG2a and IgG3 Abs and serum IL-17 and increased Th2-related anti-dsDNA IgG1 Ab, serum IL-10, and IL-4. Furthermore, the therapeutic effects of SM934 on NZB/W F1 mice were tightly linked to enhancing IL-10 production from macrophages, which was absent in MRL/lpr mice.

In another mouse model of lupus BXSB, dihydroartemisinin was found to significantly improve lupus nephritis, reduce serum TNF*α* level, and suppress TNF*α* production from peritoneal macrophages [34].

### 3.4. Artemisinin Derivatives Treat Multiple Sclerosis

Multiple sclerosis (MS) is a class of autoimmune disease occurring in the central nervous system, which was mediated by both of Th17- and Th1-type T cells. The main symptom of patients with MS was progressive paralysis. Experimental allergic encephalomyelitis (EAE) is a well-established murine model to study the pathogenesis of MS and for drug screening. Several artemisinin derivatives were reported to treat EAE including SM933, SM934, and dihydroartemisinin (DHA) with different mechanisms [8, 12, 35]. In 2007, SM933 was reported to possess unique anti-inflammatory properties through regulation of signaling pathways, leading to amelioration of EAE [35]. The anti-inflammatory properties of SM933 were characterized by a regulatory mechanism involving the NF*κ*B and the Rig-G/JAB1 signaling pathways. Regulation of the Rig-G/JAB1 pathway by SM933 led to altered cell cycle activity of encephalitogenic T cells as a result of its selective effect on activated, but not resting, T cells.

In contrast to SM933, both SM934 and DHA were demonstrated to treat EAE majorly through regulating the balance between effector T cells and regulatory T cells. Zhao et al. found that administration of DHA significantly decreased effector CD4 T cells but increased Tregs in EAE mice [8]. Their study argued that DHA reciprocally regulates effector T cell and regulatory T cell generation through modulating mTOR pathway. On top of DHA work, through both BrdU incorporation strategy and* in vitro* Treg differentiation assays, Zuo group in SIMM further revealed that SM934 treatment could directly promote the expansion of Treg cells* in vivo* and* in vitro* [12].

### 3.5. Artemisinin Derivatives Treat Allergic Diseases

Allergy is an acquired hypersensitivity reaction of the immune system mediated by cross-linking of allergen-specific IgE with high-affinity IgE receptors, leading to immediate mast cell degranulation. Allergic disorders have substantially different pathogenesis with autoimmune diseases. However, artemisinin derivatives were also reported to be therapeutic against the allergic diseases [36–38]. Several studies by Wong group demonstrated that artesunate ameliorates experimental allergic airway inflammation probably via negative regulation of PI3K/Akt pathway and blocking IgE-induced mast cell degranulation. A recent study also reported that artesunate could suppress the proliferation of airway smooth muscle, which further strengthened the reasonability for artesunate to treat allergic airway disorders.

## 4. Structure-Activity Relationship

It is broadly accepted that artemisinin exerts antimalarial effects through the following molecular mechanisms: Heme or free iron will break the peroxide bridge, which results in the degradation of the molecular structure of artemisinin to form the nucleophilic radical metabolites. Consequently, the free radicals, acting as an alkylating agent, will attack macromolecular bearing electrophilic groups or centers, which eventually leads to cell damage [17]. One elegant study showed that artemisinin inhibits endoplasmic reticulum Ca^2+^-ATPase (SERCA), resulting in calcium to accumulate in the cytoplasm [39]. The high concentration of cytoplasmic calcium activates a secondary influx of calcium into the cell, which induces the apoptosis. Thapsigargin (TG), a specific inhibitor of SERCA, could induce intracellular calcium accumulation and leads to cell apoptosis. TG is structurally similar to artemisinin, which allows it to antagonize the inhibition of artemisinin activities on SERCA* in vitro*. There are further evidences showing that TG and artemisinin have the same binding site [17]. Sequence alignments of the thapsigargin-binding pocket of mammalian SERCAs and from different* Plasmodium* spp. show several amino acids that differ among SERCAs. Further structural biology study demonstrated that a single amino acid (Leu263) in transmembrane segment 3 of SERCAs can determine susceptibility to artemisinin. Introduction of a residue in* Plasmodium vivax* SERCA (PvSERCA) increased sensitivity to artemisinins by threefold, whereas introduction of a residue in* Plasmodium berghei* SERCA (PbSERCA) decreased sensitivity by threefold [39, 40].

Although peroxide bridge plays a necessary role in the biological activity of artemisinin, finding in stereochemistry indicates that the binding site of artemisinin and SERCA does not include the peroxide bridge [41]. We hypothesize that peroxide bridge might act as a “catalyst” for artemisinin to inhibit SERCA. According to stereochemistry and transitional state theory, when the peroxide bridge is intact, the spatial configuration of artemisinin is relatively rigid, and the sesquiterpene lactone structure might not be able to flexibly rotate and fold. In this case, artemisinin has relatively lower affinity to SERCA. However, once the peroxide bridge has been reduced by divalent iron ion and broken, the sesquiterpene lactone part will be released and will be flexible and will bind to SERCA with high affinity. As a result, the inhibitory effect of artemisinin against SERCA is enhanced.

Studies have found that, in parasites-infected red blood cells and activated lymphocytes, the divalent iron ion level is significantly higher than the resting state/cells. In this case, the opportunity for peroxide bridge to be broken is largely increased, which consequently makes the activated cells, rather than the resting cells, much more vulnerable to artemisinin. In conclusion, the peroxide bridge is a unique structure and is essential for the biological activity of artemisinin. Furthermore, since mammalian SERCAs are not susceptible to inhibition by artemisinins [39], the biochemical mechanism and molecular target of artemisinin to exert immunosuppressive function still need to be further studied.

## 5. Summary and Future Outlook

**Figure 2:**
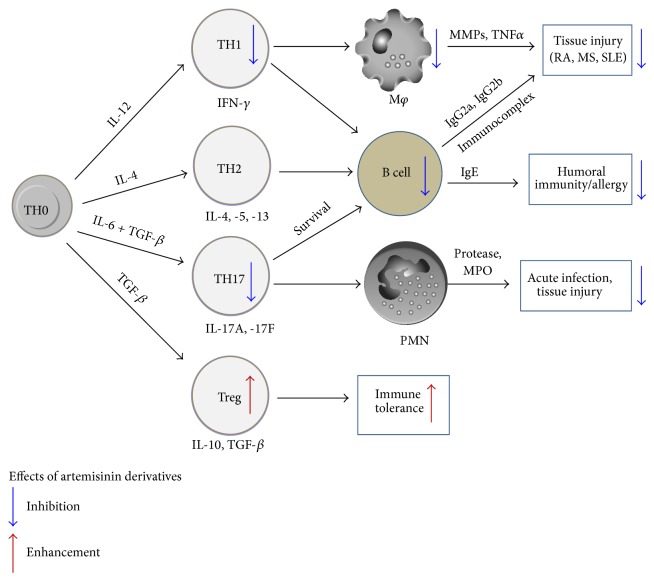
Schematic of artemisinin derivatives affecting immune and inflammation.

Artemisinin and its derivatives are potent antimalarial agents with high efficacy and low toxicity. Besides the outstanding antimalarial activity, artemisinin and its derivatives also possess immunosuppressive activities and are experimentally used to treat autoimmune diseases, such as SLE, RA, and CIA. In this paper, we summarized the recent progress of artemisinin derivatives in treating autoimmune and allergic disorders. We conclude that artemisinin derivatives perform immunosuppressive functions primarily through inhibiting pathogenic T cell activation, suppressing B cells activation and antibody production, and expanding regulatory T cells (impact of artemisinins and derivatives on different immune cells is shown in Figure 2). We deem that, as anti-inflammatory agents, artemisinin derivatives possess more advantages to act on multiple checkpoints within the immune signaling cascade, with selectivity for activated pathogenic T cells, to create a synergistic treatment effect on disease activity. Thus, these new artemisinin derivatives may be a kind of promising candidates to treat inflammation and autoimmune disorders.

## Abbreviations

DHA: Dihydroartemisinin
SM735: 3-(12-*β*-Artemisininoxy) phenoxyl succinic acid
SM905: 1-(12-*β*-Dihydroartemisinoxy)-2-hydroxy-3-tert-butylaminopropane maleate
SM933: Ethyl 2-[4-(12-*β*-artemisininoxy)] phenoxylpropionate
SM934: 2′-Aminoarteether (*β*) maleate
SLE: Systemic lupus erythematosus
RA: Rheumatoid arthritis
MS: Multiple sclerosis
EAE: Experimental allergic encephalomyelitis
CIA: Collagen-induced arthritis
MOG: Myelin oligodendrocyte glycoprotein
SERCA: Endoplasmic reticulum Ca^2+^-ATPase
TCR: T cell receptor
STAT: Signal transducer and activator of transcription
CD: Cluster of differentiation
Th: T helper
IL: Interleukin
IFN: Interferon
ConA: Concanavalin A
SERCA: Endoplasmic reticulum Ca^2+^-ATPase
Treg: Regulatory T cell
TNF: Tumor necrosis factor
TG: Thapsigargin.
